# HIV continuum of care among trans women and *travestis* living in São Paulo, Brazil

**DOI:** 10.11606/s1518-8787.2020054002374

**Published:** 2020-11-12

**Authors:** Aline Borges Moreira da Rocha, Cláudia Barros, Igor Prado Generoso, Francisco I. Bastos, Maria Amélia Veras

**Affiliations:** I Faculdade de Ciências Médicas da Santa Casa de São Paulo Departamento de Saúde Coletiva São PauloSP Brasil Faculdade de Ciências Médicas da Santa Casa de São Paulo. Departamento de Saúde Coletiva. São Paulo, SP, Brasil; II Universidade Católica de Santos SantosSP Brasil Universidade Católica de Santos. Programa de Pós-Graduação em Saúde Coletiva. Santos, SP, Brasil; III Fundação Oswaldo Cruz Instituto de Comunicação e Informação em Ciência e Tecnologia em Saúde Rio de JaneiroRJ Brasil Fundação Oswaldo Cruz. Instituto de Comunicação e Informação em Ciência e Tecnologia em Saúde. Rio de Janeiro, RJ, Brasil

**Keywords:** Transgender Persons, Transvestism, HIV Infections, prevention & control, HIV Seroprevalence, Anti-Retroviral Agents, therapeutic use, Health Services Accessibility

## Abstract

**OBJECTIVE:**

To examine the HIV care cascade among trans women and *travestis* in São Paulo – Brazil, the most populous city in South America.

**METHODS:**

Using data from a cross-sectional study carried out between November 2016 and May 2017 in the city of São Paulo (Divas Research). Respondent driven sampling (RDS) was used to recruit 386 transgender women and *travestis* who participated in a HIV risk survey and were tested for HIV. The cascade was defined as HIV prevalence, HIV diagnosed, Antiretroviral (ART) Prescription, and currently on ART. A multiple analysis model was conducted to identify the association between sociodemographics and the cascade gaps.

**RESULTS:**

Of the trans women living with HIV, 80.9% were already diagnosed, 76.6% of them had been prescribed, of which 90.3% were currently on treatment. Those who were registered in care had a higher rate of ART (aPR 2.06; 95%CI 1.09-3.88). Trans women between 31-40 years old (aPR 1.65; 95%CI 1.09-2.50) and those older than 40 (aPR 1.59; 95%CI 1.04-2.43) had higher prevalence of ART.

**CONCLUSIONS:**

Our data suggest an increase in the testing and treatment policy implementation among trans women in the city of São Paulo, although gaps have been found in the linkage to care. However, young trans women and those not registered in health care service may benefit from efforts to engage this part of the population in care to improve HIV treatment and care outcomes.

## INTRODUCTION

Among the different populations at high risk of HIV infection, people who identify as transgender women and *travestis* have one of the highest HIV infection rates around the world ^1^ . Due to gender non-conformity (non-recognition of the gender assigned at birth as their gender identity) and the challenge of persuading the society this is a valid identity to be fully respected, transgender women and *travestis* are constantly stigmatized, experience difficulties in their insertion in different institutions and contexts, including health services ^2^^–^^4^ .

In Brazil, different modalities of discrimination and prejudice, such as transphobia and unprotected sex, place this population at a higher risk of HIV/AIDS and other sexually transmitted diseases ^5^ . A study conducted in 2016 with transgender women in Rio de Janeiro identified a difference between the reported and confirmed HIV prevalence (24.2% and 31.2%, respectively), suggesting that a part of the population did not know their serological status, or did not have access to prior HIV testing before participating in the study ^6^ . Furthermore, the prevalence of HIV infection in transgender women is not documented in a systematic way by health services, neither by most surveillance systems (which frequently do not report the category “transgender”), resulting in a lack of information about the epidemic. Gathering specific epidemiological data about trans women and *travestis* can help identify the magnitude of this epidemic, its trends, and allow for the collaboration with executive health plans and reformulation of properly tailored public policies ^7^^,^^8^ .

An important mechanism to assess the profile of HIV infection in different countries is the HIV Care Continuum Cascade, a tool that uses simple mathematical modeling to illustrate a chain of events from the awareness of HIV infection to the final objective, HIV health care, which is used to get the undetectable viral load ^9^^,^^10^ .

The construction of a HIV care cascade is important to identify gaps that limit access to diagnostic and treatment services, since there is limited information on the health care of transgender women living with HIV. Considering the stigmatization and prejudice this population lives, it is imperative to analyze the features of the epidemic, produce data on the number and proportion of people living with HIV, and access treatment services and their results to evaluate the health care offered to this population.

In Brazil, there is a shortage of specific research on this population group, especially *travestis* , and their knowledge about health issues. This study aimed to analyze the HIV care cascade for trans women and *travestis* living in São Paulo, Brazil and to identify factors associated with the major gaps in the care continuum.

## METHODS

The data of this study were collected from the project “Study of national coverage of HIV, behaviors, attitudes, practices and prevalence of syphilis, hepatitis B and C between *travestis* (Divas Research),” conducted between November 2016 and May 2017 in the city of São Paulo.

A total of 386 transgender women and *travestis* were recruited using Respondent-Driven Sampling (RDS), method that relies on social networks, with participants inviting other members from their population to be enrolled in the research. Five “seeds” (first participants) were selected from São Paulo, which were identified in a previous formative research. At the field, each participant was oriented on the inclusion criteria, reading and signing the informed consent form (ICF). Additionally, each participant received three invitations, which initiate the recruitment chains. This sampling method assumes that members of the targeted community are more adept at locating and recruiting other participants ^11^ . Monetary support was given to the participants for transportation and recruitment expenses: each participant received $10 after their first interview, and they could potentially receive an additional $10 for each referral that participates in the research. More details about the methodology can be found in a previous article ^12^ . After the interview, participants are quickly tested for HIV and other sexual transmitted infections (STI). Any person with a positive result is immediately linked to care to proceed diagnostics and appropriate treatment, when applicable.

Trained interviewers applied structured questionnaires, which were used for data collection. Sociodemographic factors assessed in this study were gender identity, skin color, education level, income, age, housing situation, relationship status, and health care service registration. The variable “housing situation” considered the type of housing, classifying participants as “stable” (homeowners, renting, or living with relatives) or “unstable” (hotel, occupation, shelter, or living in their workplace). The variables composing sociodemographics represent what could interfere with the outcomes related to HIV infection and treatment, along with those presented in the Brazilian Ministry of Health report of clinical monitoring of HIV in Brazil ^13^ .

The construction of the HIV care cascade was conducted using variables available in the questionnaire that correspond with those used in classic models of cascades ^9^ . The variables chosen were the proportion of trans women living with HIV (HIV prevalence), proportion of trans women previously aware of their serological status (HIV diagnosed), proportion of Antiretroviral (ART) prescription, and proportion of current adherence to ART (medication intake). This study did not have access to the viral load count of the participants, and it did not include a specific question that could measure retention in care.

In the statistical analysis, as assumed in RDS sampling procedures, a weight for each participant was defined using the estimator RDS II from the software RDS Analyst version 12. Descriptive statistics, association tests, and mathematical models were performed using complex sampling design procedures, which consider the secondary dependence to the underlying chain-referral process. Categorical variables were described by relative frequencies and respective confidence intervals. Bivariate analysis was used to identify factors associated with the gaps of HIV care cascade using Poisson regression ^14^ . In the multiple analysis model, we included variables with p < 0.20 and those found in previous literature. A 5% significance level was considered. The analysis considered the sampling design and was done in Stata, version 13.

The study protocol was reviewed and approved by Sergio Arouca National School of Public Health (ENSP/FIOCRUZ) Research Ethics Board (CAAE-49359415.9.0000.5240). All participants provided written informed consent, participants could withdraw consent at any stage of the process or skip any questions or laboratorial testing if considered it too personal, sensitive or distressing.

## RESULTS

Our study enrolled a total of 386 participants. Most of them self-identified as *travesti* (50.5%) and trans women (39.9%). Most of our sample was composed of adults from 25 to 40 years, and 23.6% were classified as youth (24 years or younger). Skin color was also self-declared, 50.5% of the participants identified as mixed race and 15.9% identified as black. The data collected for educational status indicated that almost half of the respondents had some high school (47.5%) and only 12% had attended a university or college. Information collected regarding income indicated that 36.3% of the participants earned less than 242.22 US dollars per month, which constitute the minimum wage in Brazil. Additionally, 30.4% of the participants reported unstable housing. Most participants were single (62.8%) and registered in primary health care services (80.5%) ( Table 1 ).

**Table 1:** Sociodemographic characteristics of trans women and *travestis* living in São Paulo, Brazil, 2017 (n = 386)

Variable		% *	95%CI
Gender			
	Woman	9.32	5.93–14.37
	Trans woman	39.95	32.95–47.73
	Travesti	50.5	42.94–58.03
Skin color			
	White	30.02	23.38–37.61
	Black	15.91	11.43–21.71
	Mixed	50.55	42.99–58.09
	Other (Indigenous/Yellow)	3.53	1.77–6.89
Education			
	More than high school	12.04	7.63–18.47
	High school	40.41	33.17–48.09
	Less than high school	47.55	40.09–55.13
Income (minimum wage - MW)			
	More than three MW	14.74	10.06–21.09
	Between one and three MW	48.95	41.43–56.53
	Less than one MW	36.31	29.52–43.68
Age			
	< 24	23.62	17.82–30.61
	25–30	32.39	25.65–39.95
	31–40	29.05	22.61–36.47
	> 40	14.94	10.47–20.86
Housing situation			
	Stable	69.61	62.56–75.85
	Unstable	30.39	24.15–37.44
Relationship status			
	Single	62.82	55.08–69.95
	Dating	14.63	9.86–21.17
	Married	21.62	15.82–28.82
	Other	0.93	0.29–2.96
Registered in health care service			
	No	19.51	13.87–26.72
	Yes	80.49	73.28–86.13

* Weighted proportion using RDS complex sampling procedures.

A total of 148 participants tested positive for HIV, indicating a 38% (95%CI: 30.9-45.6) of prevalence, of which 80.9% (95%CI: 67.2-89.7) had been previously diagnosed and were aware of their positive status. Of this, 76.6% (95%CI: 61.6-87.0) had received ART prescription, and 90.8% of those (95%CI: 82.0-95.5) reported currently taking ART. ( Figure 1 ).

**Figure:**
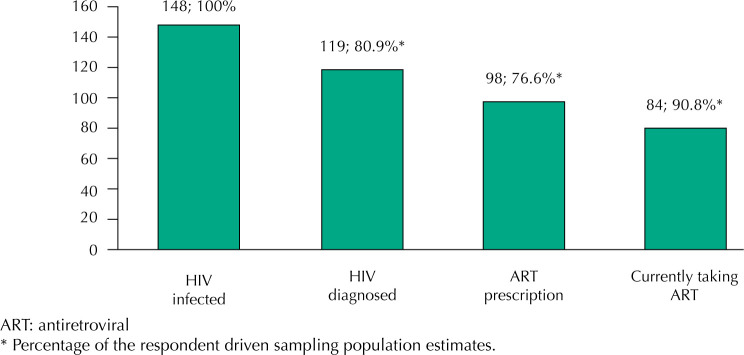
HIV care cascade among trans women living with HIV in São Paulo, Brazil (n = 148).

The total of 80.5% participants were registered in health care services, among them 88.2% were aware of their positive status, 78.9% received ART prescription, and 94.3% were currently on ART at the time of the survey. We observed that only 64.6% of youth were taking ART, while among all other age brackets this proportion was higher than 90% ( Table 2 ).

**Table 2:** Sociodemographic characteristics by HIV diagnosed, antiretroviral (ART) prescription and currently taking ART (row) for trans women in São Paulo, Brazil.

Variable		HIV diagnosed		p	ART prescription		p	Currently taking ART		p
		% *	95%CI		% *	95%CI		% *	95%CI	
Gender				0.382			0.675			0.393
	Woman	59.0	20.6–88.9		86.1	50.3–97.4		76.8	30.4–96.2	
	Trans woman	86.4	60.6–96.3		72.3	47.2–88.5		94.5	76.4–98.9	
	*Travesti*	80.6	60.9–91.8		79.4	58.3–91.4		89.8	76.8–95.9	
Skin color				0.697			0.691			0.134
	White	75.8	43.5–92.8		84.6	43.5–97.5		94.7	79.4–98.8	
	Black	89.0	63.8–97.4		72.3	43.6–89.8		100	-	
	Mixed	81.1	62.7–91.7		73.5	52.9–87.3		89.0	75.6–95.5	
	Other (Indigenous/Yellow)	90.8	62.8–98.3		100	-		56.5	11.9–92.5	
Education				0.968			0.745			0.694
	More than high school	76.9	27.7–96.6		69.9	20.9–95.3		87.5	43.4–98.5	
	High school	81.3	54.5–94.0		83.3	56.2–95.1		94.2	79.6–98.6	
	Less than high school	81.5	63.3–91.9		73.5	52.8–87.3		88.8	74.4–95.5	
Income (minimum wage - MW)				0.061			0.394			0.082
	More than three MW	50.8	21.4–79.8		95.8	85.4–98.9		100	-	
	Between one and three MW	82.7	58.5–94.3		74.7	49.2–90.1		96.9	86.5–99.4	
	Less than one MW	89.0	69.7–96.6		74.7	52.7–88.7		83.3	66.8–92.6	
Age				0.098			0.159			**0.002**
	< 24	69.5	38.5–89.3		75.9	48.2–91.5		64.6	33.4–86.9	
	25–30	74.3	48.3–89.9		71.6	43.1–89.4		93.4	75.8–98.8	
	31–40	89.8	74.7–96.3		62.1	30.9–85.7		99.0	92.7–99.8	
	> 40	98.5	89.3–89.8		100	-		100	-	
Housing situation				0.871			0.760			0.260
	Stable	79.9	60.9–91.1		74.8	53.1–88.7		93.8	83.4–97.8	
	Unstable	81.8	58.7–93.5		78.8	56.4–91.5		86.6	68.9–94.9	
Relationship status				0.932			0.680			0.474
	Single	80.6	61.9–91.4		80.0	62.4–90.7		93.8	84.4–97.7	
	Dating	77.6	41.6–94.4		80.2	33.6–97.0		89.9	62.2–97.9	
	Married	83.6	48.6–96.5		64.9	31.1–88.4		82.5	49.9–95.7	
	Other	100	-		84.2	32.4–98.3		87.5	36.1–98.8	
Registred in health care service				**0.003**			0.255			< 0.001
	No	45.1	17.5–76.0		55.3	16.6–88.5		43.4	13.8–78.5	
	Yes	88.2	75.2–94.8		78.9	63.3–89.0		94.3	85.9–97.8	

* Weighted proportion using RDS complex sampling procedures. Bolded values: Statistically significance.

No statistically significant association was found between the awareness of HIV infection and ART prescription. In the bivariate analysis, no statistically significant association was found between any sociodemographic characteristics and ART prescription. Also, in bivariate analysis we found that individuals who receive less than the minimum wage as income had a lower prevalence ratio of currently taking antiretroviral medication (PRR 0.83; 95%CI:0.71-0.97) ( Table 3 ).

**Table 3:** Univariate and multivariate analysis for having antiretroviral (ART) prescription and ART uptake among trans women in São Paulo, Brazil, 2017.

Variable		Have ART prescription				Currently take ART			
		PRR	95%CI	aPRR	95%CI	PRR	95%CI	aPRR	95%CI
Gender									
	Woman	Reference	-	Reference	-	Reference	-	Reference	-
	Trans woman	0.84	0.56–1.24	**0.56**	**0.31–0.98**	1.23	0.76–1.98	1.16	0.90–1.49
	Travesti	0.92	0.66–1.27	0.64	0.37–1.11	1.16	0.72–1.88	1.15	0.90–1.47
Skin color									
	White	Reference	-	Reference	-	Reference	-	Reference	-
	Black	0.85	0.54–1.34	0.73	0.51–1.05	1.05	0.97–1.14	0.99	0.86–1.14
	Mixed	0.86	0.59–1.27	0.89	0.68–1.15	0.93	0.82–1.07	0.97	0.85–1.11
	Other (Indigenous/ Yellow)	1.18	0.87–1.59	1.50	0.91–2.45	0.59	0.22–1.59	0.98	0.51–1.87
Education									
	More than High School	Reference	-	Reference	-	Reference	-	Reference	-
	High School	1.19	0.59–2.37	1.00	0.66–1.54	1.07	0.80–1.43	1.12	0.86–1.47
	Less than High School	1.05	0.52–2.10	0.79	0.51–1.24	1.01	0.75–1.36	1.02	0.75–1.39
Income (minimum wage - MW)									
	More than three MW	Reference	-	Reference	-	Reference	-	Reference	-
	Between one and three MW	0.78	0.58–1.04	0.78	0.58–1.04	0.96	0.92–1.01	0.96	0.81–1.14
	Less than one MW	0.77	0.60–1.00	0.66	0.43–1.03	**0.83**	**0.71–0.97**	**0.81**	**0.67–0.98**
Age									
	< 24	Reference	-	Reference	-	Reference	-	Reference	-
	25–30	0.94	0.60–1.47	0.96	0.62–1.48	1.44	0.90–2.30	1.34	0.92–1.94
	31–40	0.81	0.46–1.44	0.81	0.48–1.37	1.53	0.96–2.42	**1.65**	**1.09–2.50**
	> 40	1.31	0.98–1.76	**1.56**	**1.06–2.30**	1.54	0.97–2.44	**1.59**	**1.04–2.43**
Housing situation									
	Stable	Reference	-	Reference	-	Reference	-	Reference	-
	Unstable	1.05	0.76 −1.46	1.17	0.82–1.66	0.92	0.78–1.08	1.03	0.91–1.17
Relationship status									
	Single	Reference	-	Reference	-	Reference	-	Reference	-
	Dating	1.00	0.64–1.56	1.11	0.64–1.93	0.95	0.79–1.14	1.05	0.82–1.35
	Married	0.81	0.47–1.37	0.96	0.67–1.38	0.87	0.66–1.16	0.85	0.70–1.03
	Other	1.05	0.69–1.59	1.22	0.62–2.40	0.93	0.67–1.28	0.82	0.57–1.16
Registred in health care service									
	No	Reference	-	Reference	-	Reference	-	Reference	-
	Yes	1.42	0.62–3.27	1.63	0.83–3.17	2.17	0.89–5.28	**2.06**	**1.09–3.88**

Bolded values: Statistically significance.

In the multivariate regression, individuals who self-identify as trans women have lower adjusted prevalence ratio of having ART prescription (aPR 0.56; 95%CI: 0.31-0.98) when compared with those who self-identify as women. Furthermore, the older individuals (more than 40 years old) had a higher adjusted prevalence ratio of having ART prescription (aPR 1.56; 95%CI: 1.06-2, 30). According to multivariate analysis, age groups between 31-40 (aPR 1.65; 95%CI: 1.09-2.50) and older than 40 years old (aPR 1.59; 95%CI:1.04-2.43) presented higher adjusted prevalence ratio of taking the medicine. Additionally, receiving less than the minimum wage of income negatively affects ART usage (aPR 0.81; 95%CI: 0.67-0.98). Being registered in health care services had a higher adjusted prevalence ratio of having ART prescription when compared with not being registered (aPR 2.06 95%CI: 1.09-3.88) ( Table 3 ).

## DISCUSSION

Over a third of trans women and *travestis* in our sample are living with HIV, which is similar to what was found in another study conducted in a different Brazilian city, Rio de Janeiro, that reported a similar HIV prevalence (31.2%) for this population ^7^ . A recent meta-analysis estimated a worldwide HIV prevalence of 19.1% among transgender women ^1^ . The results of our study indicate a higher HIV prevalence for trans women and *travestis* in São Paulo compared with the pooled worldwide data.

The HIV care cascade indicators show a suboptimal value of HIV infection diagnosis but a slightly better figure of engagement in medical treatment according to the 90-90-90 goals ^15^ . Our results of 80.9% of HIV diagnosed and 90.8% of trans women currently on ART are better than those found in the Brazilian National Cascade, in which 84% of the general population living with HIV is diagnosed and 63% is currently taking ART ^13^ . One must remark that national data summarize information from a heterogeneous pool of specialized/referral services as well as services providing care and management for a broad set of people living with AIDS. On the other hand, our study deals with data originated exclusively from participants living in the city of São Paulo, located in the state of São Paulo, which is consistently showing better indicators, compared with other Brazilian states, being able to maintain a dedicated and multi-professional team engaged in data collection and care provision.

In our study no statistically significant association was found between the sociodemographic factors and HIV awareness, suggesting that these factors may not interfere individually with the diagnosis in our sample, whereas other study found that factors such as young age negatively interfered with HIV testing, with people under 18 years old having less chance of being tested when compared with the older ones ^5^ . Alternatively, our sample may lack statistical power to indicate some potential associations or may be biased by bottlenecks and their associate design effects that may be relevant on any chain-referral based-study ^16^ .

The results presented in this study may be one of the positive consequences of the national public policy established in 2013 addressing the health care needs of the LGBT population, which contributed to the implementation of testing services and campaigns for the transgender population at national and local levels ^17^ . This policy is focused on preventive actions for groups affected by a high burden of HIV infection in Brazil, including campaigns aiming to reduce stigma and dedicated services available to trans women, scaling up HIV testing offered by public services and by non-governmental organizations. Such changes may have led to an increase in HIV testing by the transgender population in São Paulo, but these results do not necessarily were translated into a greater referral for treatment services ^18^ . It is too soon to ascertain the effect of broad interventions on a population facing long-entrenched prejudice and stigma.

The major gap in our cascade is related to ART prescription. We observed that 23.4% of the trans women in São Paulo who were diagnosed with HIV reported not having ART prescription, that is, they did not receive medical care. This result may show a failure in the linkage to care; however, it is not a direct measure of this phenomenon. Note that the absence of prescription is not exclusively related to the difficulty in accessing medical services, but also to the low retention at health services as a consequence of stigma and discrimination faced by this population, especially the ones related to HIV ^19^ .

In the multivariate analysis, individuals who self-identified as trans women were less likely to receive ART prescription when compared with those who self-identify as women ^20^ . This may reflect the individual's capability to appear and to be treated as her preferred gender, protecting them of being discriminated. It is known that gender-affirming care and the use of correct pronouns can improve health and quality of life for transgender people ^21^ .

The negative effects of HIV on this population, including the HIV transmission may be reduced with appropriate medical care, respecting gender identity, and introducing an early antiretroviral therapy ^22^ .

Traditional cascades do not rely on ART prescription in its construction but assessing this variable may be useful when dealing with stigmatized populations, as a proxy of individual's attendance to a consultation for HIV treatment.

Furthermore, older individuals are more likely to receive prescription and take the medication in comparison to the younger ones. This result is similar to HIV care continuum cascades for general Brazilian population, in which older individuals present higher rates of ART uptake ^13^ . A qualitative study conducted with transgender youth, presented several barriers to access HIV services and to start ART treatment, such as unstable socioeconomic situation, fear of lack of confidentiality, low awareness of available services, and societal oppression ^23^ . In another care cascade of transgender women from San Francisco, similar association between age and enrollment in HIV care was found ^24^ . These data indicate the need for policies and strategies that improve the access to treatment for young transgender women, probably in their gender transition phase, considering that age seems to be a determinant factor in HIV care for this population.

More than 90% of our sample is currently on ART, suggesting we are reaching targeted values to the 90-90-90 goals. This indicates that a significant proportion of trans women and *travestis* in the city of São Paulo have access to HIV care; however, there is a lack of information in the process of linkage to services. We found that trans women who receive less than the minimum wage (< U$250/month) had fewer chances of being currently on ART, illustrating the effect of socioeconomic situation on transgender health.

Thus, people registered to health care services presented a statistically significant association with medication uptake, showed by a greater prevalence ratio of using ART for trans women registered in a health services and by the fact that more than 90% of the trans women who take the medication are enrolled in health services. Since in Brazil HIV medications are only dispensed by the Unified Health System (SUS), which requires the registration of users in specialized HIV/AIDS care services, HIV treatment is closely associated with being enrolled in public health services ^18^ . Therefore, it is not possible to use this finding to document that trans women are officially bounded to health services, but it is possible to infer that they are accessing one of the available gateways to health care.

As a cross-sectional study, it is impossible to assess the directionality of associations. This study neither quantified the viral load of the participants nor the information about linkage to care in the questionnaire, which compromise the optimal construction of the classic care continuum cascade. Notwithstanding, there is a lack of studies analyzing care cascades among trans women in the literature. This is the first study to design a care continuum for trans women and *travestis* in São Paulo, the largest city in Latin America, and the second study of its kind in Brazil.

The results from this study suggest that an increase in HIV testing and the implementation of policies aimed to enhance the treatment among trans women and *travestis* in São Paulo city are sorely necessary to offer an acceptable, humane, and quality healthcare to this population. Young trans women and those not registered in health care services may benefit from multi-level efforts to engage this part of the population in care, providing health services that meet the demands of this population and be prepared to receive it, improving HIV care outcomes.
